# Mechanical feedback coordinates cell wall expansion and assembly in yeast mating morphogenesis

**DOI:** 10.1371/journal.pcbi.1005940

**Published:** 2018-01-18

**Authors:** Samhita P. Banavar, Carlos Gomez, Michael Trogdon, Linda R. Petzold, Tau-Mu Yi, Otger Campàs

**Affiliations:** 1 Department of Physics, University of California, Santa Barbara, Santa Barbara, California, United States of America; 2 California NanoSystems Institute, University of California, Santa Barbara, Santa Barbara, California, United States of America; 3 Department of Molecular, Cell and Developmental Biology, University of California, Santa Barbara, Santa Barbara, California, United States of America; 4 Department of Mechanical Engineering, University of California, Santa Barbara, Santa Barbara, California, United States of America; 5 Center for Bioengineering, University of California, Santa Barbara, Santa Barbara, United States of America; Purdue University, UNITED STATES

## Abstract

The shaping of individual cells requires a tight coordination of cell mechanics and growth. However, it is unclear how information about the mechanical state of the wall is relayed to the molecular processes building it, thereby enabling the coordination of cell wall expansion and assembly during morphogenesis. Combining theoretical and experimental approaches, we show that a mechanical feedback coordinating cell wall assembly and expansion is essential to sustain mating projection growth in budding yeast (*Saccharomyces cerevisiae*). Our theoretical results indicate that the mechanical feedback provided by the Cell Wall Integrity pathway, with cell wall stress sensors Wsc1 and Mid2 increasingly activating membrane-localized cell wall synthases Fks1/2 upon faster cell wall expansion, stabilizes mating projection growth without affecting cell shape. Experimental perturbation of the osmotic pressure and cell wall mechanics, as well as compromising the mechanical feedback through genetic deletion of the stress sensors, leads to cellular phenotypes that support the theoretical predictions. Our results indicate that while the existence of mechanical feedback is essential to stabilize mating projection growth, the shape and size of the cell are insensitive to the feedback.

## Author summary

All morphogenesis processes, whether at the cell scale or tissue level, require the coordination of growth and mechanics to properly shape functional structures. However, the mechanisms that coordinate these two processes in the sculpting of individual cells, and especially in walled cells, remain unknown. Using yeast mating projection growth as a model system, we show that a genetically-encoded mechanical feedback relays information about the mechanical state of the cell wall to the intracellular processes assembling it, thereby coordinating cell wall expansion and growth during cell morphogenesis. We find that mechanical feedback is essential to stabilize cell growth, but the shape and size of the cell are insensitive to the feedback and independently controlled.

## Introduction

From cell division to polarization and growth, cells constantly change their shapes to perform specific tasks [1–3]. These morphological changes are achieved through remodeling of the structures that mechanically sustain the cell, such as the cytoskeleton in animal cells and the cell wall in walled cells. Unlike animal cells, which can undergo fast and complex cell shape changes, walled cells must take extra care during shape changes, as the cell wall needs to mechanically sustain their high internal turgor pressure throughout the cell wall remodeling process [4–6]. A lack of coordination between cell wall expansion and assembly during cell growth can be fatal for the cell, as the thinning of the cell wall in expanding regions may lead to cell lysis unless it is carefully balanced by newly assembled wall material. While it is believed that the coordination of cell wall expansion and assembly is necessary to cell wall remodeling and morphogenesis, the mechanisms behind this coordination remain largely unknown.

**Fig 1:**
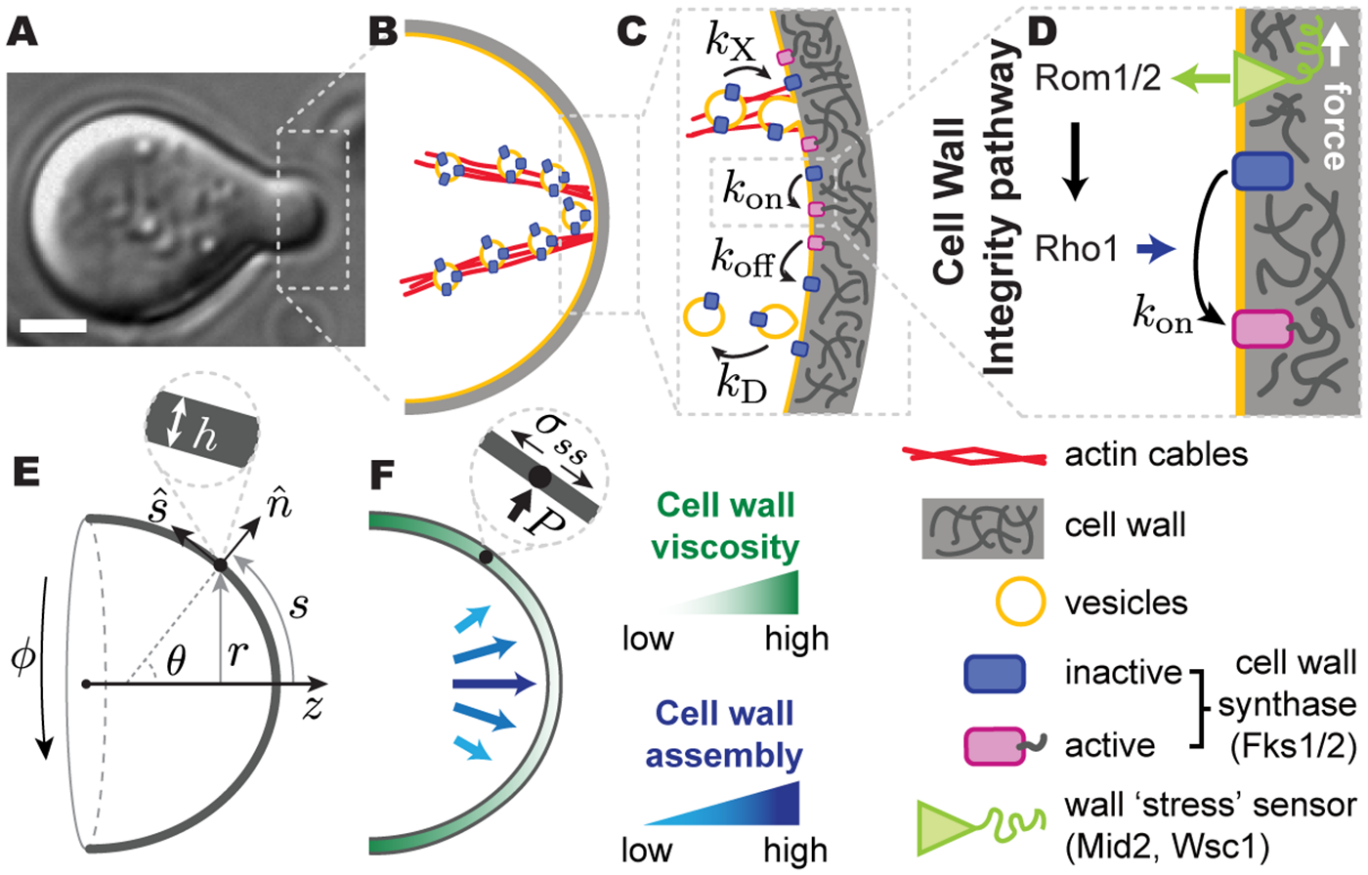
Schematic diagrams of relevant events and quantities in mating projection growth. (A) Transmitted light image of a *S. cerevisiae* cell growing a mating projection in the presence of *α*-factor. Scale bar, 2*μm*. (B-D) Sketch of molecular events leading to the delivery and activation of cell wall synthases Fks1/2 at the apex. See main text and Table 1 for definitions of parameters. (E) Geometry of the system and definition of the relevant variables. (F) Sketch depicting the increasing cell wall viscosity and decreasing cell wall assembly away from the apex. The inset depicts local normal force balance at the cell wall. All variables are defined in the main text.

Cell shape changes are ultimately governed by the mechanical state of the cell wall [5–7]. Studies of the mechanics of walled cell morphogenesis have predominantly focused on tip-growing cells of plant and fungal species because of their large size, simpler geometry and fast growth rates [8–10]. In this highly polarized growth mode, cells adopt a tubular shape that extends only at the apical region (Fig 1). During this process, cells polarize their cytoskeleton and localize exocytosis to the growing region, exactly where the cell wall needs to be assembled and remodeled. While the molecular underpinnings of tip-growth differ across species, two basic features have been shown to be necessary [7]: polarized assembly of new cell wall material at the tip, and inhomogeneous mechanical properties enabling its apical expansion (Fig 1F). Previous theoretical descriptions of tip-growth focused on cell wall assembly [11–13] or cell wall mechanics [10, 14, 15] separately. More recent descriptions accounted for both cell wall assembly and mechanics [16–19], but assumed these processes to be independent of each other. As we show below by directly solving the dynamics of cell wall assembly and expansion, assuming cell wall mechanics and assembly to be independent of each other always leads to unstable cell wall expansion and cell lysis, in stark contrast with experimental observations. Despite its relevance to cell viability during cell wall remodeling and morphogenesis, no previous theoretical descriptions have addressed the role of coordination (coupling or feedback) between cell wall mechanics and assembly in the morphogenesis of walled cells.

In addition to well-known model systems for tip-growth, such as pollen tubes in plants and hyphal growth in higher fungi [7–9], budding yeast displays tip-growth during mating. Haploid cells secrete pheromone (*α*- and **a**-factors for mating types **a** and *α*, respectively) that elicits the growth a tubular mating projection from the partner of the opposite type [20, 21] (Fig 1A). Since the molecular basis of cell polarization and cell wall assembly and remodeling have been extensively studied in budding yeast, tip-growth of mating projections provide a unique system to study the mechanism of coordination between cell wall mechanics and assembly.

In **a**-cells, binding of *α*-factor to its cognate receptor activates the heterotrimeric G-protein, leading to the activation and polarization of the small G-protein Cdc42, a master regulator of cell polarization [22]. Cdc42-mediated polarization recruits various molecular factors to an apical region of the plasma membrane known as the polarisome, where the formin Bni1 drives the nucleation of actin cables, focusing exocytosis at the apex [3, 20] (Fig 1B). Secretory vesicles transporting Fks1/2 cell wall synthases and cell wall remodeling enzymes (e.g., glucanases) move along actin cables to the exocyst, eventually leading to the incorporation of Fks1/2 synthases to the plasma membrane and the release of glucanases into the preexisting cell wall (Fig 1B and 1C) [23–26]. Together, these events molecularly and mechanically polarize the cell, causing a localized expansion of the cell wall at the apex (Fig 1B and 1F).

In general, the expansion of the cell wall is a dangerous situation that the cell needs to carefully control. Since the cell wall sustains the high cell’s internal turgor pressure, uncontrolled cell wall expansion can lead to cell wall piercing and cell lysis. In budding yeast, the Cell Wall Integrity (CWI) pathway is known to help the cell prevent loss of cell wall mechanical integrity in a variety of situations [27–30], from mating pheromone-induced growth to vegetative growth [28, 29, 31]. Five transmembrane proteins, namely Wsc1, Wsc2, Wsc3, Mid2, and Mtl1, are thought to act as stress sensors and relay information about the mechanical state of the cell wall to multiple intracellular processes via the activation of Rho1 GTPases [28, 29, 32–37]. Previous works have shown that Wsc1 and, especially, Mid2 play an important role during mating pheromone induced growth, while the remaining stress sensors do not seem to strongly affect projection growth [37–41]. While the specific mechanical quantity that these stress sensors monitor in the cell wall remains unclear, activation of the CWI pathway leads to the downstream Rho1-mediated activation of several key molecular components, including cell wall synthases (Fks1/2), actin nucleators (Bni1) and mediators of exocytosis (Sec3), and also induces a transcriptional response via a MAPK cascade [29]. The activation of cell wall Fks1/2 synthases [21, 29, 33] provides the most direct coupling between cell wall mechanics and assembly and could potentially stabilize mating projection growth (Fig 1D). However, it is unknown if such a simple, direct mechanical feedback can stabilize morphogenesis of walled cells by itself.

Using mating projection growth in budding yeast as a model system, and combining experiments and theory, we show that coordination between cell wall mechanics and assembly through direct Fks1/2 activation in the CWI pathway (mechanical feedback) stabilizes mating projection growth without affecting its geometry. In what follows, the term ‘mechanical feedback’ refers to the nature of the input signal that is sensed and relayed by stress sensors in the CWI pathway. We first derive a theoretical description that connects the cell wall mechanics to the intracellular processes building the wall (Fks1/2 activation dynamics) via the CWI pathway, and show that stable projection growth can only persist in the presence of mechanical feedback. In the absence of coordination between cell wall assembly and mechanics, cell wall expansion is always unstable, leading to either progressive thickening or thinning of the cell wall depending on conditions. Our experimental results indicate the compromising the mechanical feedback through genetic deletions of the wall stress sensors Mid2 and Wsc1, and also through perturbations of cell wall mechanics and increased turgor pressure, all lead to defects in mating projection growth and cell viability. Our experimental observations are in agreement with the theoretical predictions, suggesting that the mechanical feedback provided by the CWI pathway via direct activation of Fks1/2 synthases can stabilize projection growth without altering cell geometry. In addition, by directly measuring the size of the exocytosis region in wild-type (WT) and mutants with compromised mechanical feedback, we show that the size of the mating projection is controlled by the size of the exocytosis region, but is independent of the strength of the mechanical feedback, as predicted theoretically. Altogether, our results show that a mechanical feedback between cell wall mechanics and assembly is essential for stability of cell wall expansion and projection growth, but that its geometry and size are insensitive to the mechanical feedback.

## Materials and methods

### Numerical integration of governing equations

The system of Eqs 1–5 was scaled and written in a manner such that *r*, *h*, *ρ*_*A*_, and *ρ*_*I*_ were described by equations evolving in time, and *u*, *θ*, *κ*_*s*_ by differential equations in *s*. The latter equations were solved by the method of lines; *s* was discretized and the *s*-derivatives were written as a differential matrix using fourth order central difference and one sided finite differences at the boundary. The resulting system becomes a differential algebraic system (DAE), which was solved using Sundials, a suite of nonlinear and DAE solvers. Steady state solutions were obtained by ensuring that all time derivatives of scaled variables were below 10^−3^.

### Yeast strains and culture conditions

All yeast strains were derivatives of W303-1A and contained the *bar1Δ* mutation that prevents *α*-factor degradation by deletion of the Bar1 protease. Genetic techniques were performed per standard methods [42]. Yeast strains used in this study are listed in Table A in S1 File. All strains were cultured in YPD (yeast extract-peptone-dextrose) media supplemented with adenine. The *wsc1Δ*mid2*Δ* strain was grown in YPD media with 1M sorbitol to increase viability. Gene deletions and GFP-tagging were constructed by genomic integration using vectors amplified and targeted by PCR primers [43].

### Cell viability measurements

Cell lysis was determined by propidium iodide (Molecular Probes) staining. Propidium iodide (PI) was prepared in DMSO at a concentration of 20 mM and then diluted 1:1000 for use. Propidium iodide was added to cells after being exposed to *α*-factor (1 *μ*M) for 2 hours. To observe the viability of cells after altering the osmotic pressure, we diluted the YPD media with distilled water upon addition of propidium iodide. The cells were imaged on slides after being exposed to propidium iodide for 10 minutes. Brightfield and fluorescent (RFP filter set) images were acquired using an inverted Nikon Eclipse TE300 microscope with a 60x objective (NA = 1.4). Image analysis was manually performed using ImageJ. Data from 3 samples for each condition was averaged and, for each sample, 150 cells or more were analyzed.

### Cell lysis due to zymolyase

To decrease the viscosity of the cell wall, we utilized zymolyase, which contains *β*-1,3 glucanase, to hydrolyze the glucan linkages that strengthen the wall. Zymolyase (Zymo Research, 1 *μ*l (2 units) per 100 *μ*l of cells) was added to cells exposed to alpha-factor for 1.5 hours. Cells were treated additionally with concanavalin A to immobilize them during the imaging process. The cells were imaged on slides for 7 minutes after being exposed to zymolyase for 3 minutes. DIC images were acquired every 3 seconds. Data from 5 samples for each condition was averaged and, for each sample, 15 cells or more were analyzed. Image analysis was manually performed using ImageJ.

### Imaging and analyzing exocytosis

The length-scale of exocytosis was measured in strains that contained Sec3 fused to GFP. Calcofluor White Stain (Sigma-Aldrich) was added to cells 10 minutes prior to imaging (final concentration 0.1mg/ml) to distinguish the cell wall during image analysis. To properly visualize the length-scale and reduce imaging noise, we averaged 30 consecutive confocal images, taken at 2 second time intervals, for each cell, after incubation in 1 *μ*M *α*-factor for 1 hour and 30 minutes. For *spa2Δ* cells, the 30 images were taken at 13 second intervals to average over a longer time period to average out the stronger fluctuations in polarization in this mutant. Images were acquired with a laser-scanning confocal microscope (Zeiss LSM 710), using a 100x objective (NA = 1.4). The cells were immobilized to a glass-bottom dish coated with concanavalin A. To horizontally orient the mating projections, we layered a YPD (supplemented with 1 *μ*M *α*-factor) agarose pad on top of the cells. Image analysis was manually performed using ImageJ.

## Results

### Theoretical description

The expansion of the cell wall during morphogenesis is powered by the cell’s internal turgor pressure, *P*. Such high pressure is mechanically sustained by the cell wall, which provides mechanical integrity to the cell at all stages, including during mating projection growth. Similarly to other organisms [5, 7, 9], the cell wall in budding yeast can be considered a thin shell surrounding the cell, as the wall thickness (∼100 nm [44]) is much smaller than the radius of the projection (∼1*μm* [45]). Since the cell’s shape is determined by the location of its cell wall, we describe the growth of the mating projection as the expansion of an axisymmetric thin shell, parametrized by the arclength *s* from the projection apex and azimuthal angle *ϕ* (Fig 1E). The shape of the projection is characterized by its local radius, *r*(*s*, *t*), and the principal curvatures *κ*_*s*_ = ∂*θ*/∂*s* and *κ*_*ϕ*_ = sin*θ*/*r*, respectively, where *θ*(*s*, *t*) is the angle between the local outward normal and the axis of growth (Fig 1E). The coordinates (*r*, *ϕ*, *z*) (Fig 1E) are standard cylindrical coordinates, and the angle *θ* and arclength *s* parameterize changes in normal and tangential directions of the surface, $\hat{n}$ and $\hat{s}$ respectively [19, 46] (Fig 1E). The time evolution of the mating projection shape is governed by the mechanics and assembly of the cell wall, as described below.

#### Cell wall mechanics and extension

Building on previous work combining cell wall mechanics and growth in tip-growing cells [19], as well as on the expansion of thin viscous shells [46], we write the equations governing the dynamics of the growing cell wall. Local normal force balance at the cell wall reads

$$\sigma_{\text{ss}}\kappa_{s}+\sigma_{\phi \phi }\kappa_{\phi }=P\;\;\;\;\;\text{and}\;\;\;\;\;\sigma_{\text{ss}}\kappa_{\phi }=\frac{P}{2}\;,$$ (1)

where *σ*_*ss*_(*s*, *t*) and *σ*_*ϕϕ*_(*s*, *t*) are the tensions along *s* and *ϕ* in the wall (Fig 1F). The expansion of the cell wall during growth is caused by the tensions and depends on the mechanical properties (rheology) of the cell wall, which govern the response of the cell wall to applied stresses. Although the yeast cell wall behaves elastically at short time scales (seconds [44]), it expands irreversibly on the characteristic timescales of mating projection growth (minutes [16]), revealing a fluid-like behavior of the cell wall in growing regions. The transition between fluid-like behavior at the growing apical region to an elastic behavior far away from the apex has been studied in other systems and it is believed to be controlled by an increasing concentration of cross-links between wall polymers away from the tip [47, 48]. This is consistent with the higher concentration of cell wall degrading enzymes (glucanases) in the apical region of the mating projection [49]. We therefore assume the cell wall of the growing mating projection to behave as an inhomogeneous viscous fluid, with spatially varying viscosity *μ*(*s*), minimal at the apex and increasing away from it (Fig 1F). The local tangential velocity *u*(*s*, *t*) of a cell wall with constant density *ρ*_*w*_, or its strain (expansion) rates ${\dot{\epsilon }}_{s}=\partial u/\partial s$ and ${\dot{\epsilon }}_{\phi }=\left(1/r\right)\left(dr/dt\right)$ equivalently, can be minimally related to the tensions in the wall by [19, 46]

$$\sigma_{\text{ss}}=4\mu h[{\dot{\epsilon }}_{s}+{\dot{\epsilon }}_{\phi }/2]\;\;\;\;\;\text{and}\;\;\;\;\;\sigma_{\phi \phi }=4\mu h[{\dot{\epsilon }}_{s}/2+{\dot{\epsilon }}_{\phi }]\;.$$ (2)

#### Dynamics of cell wall assembly

Sustained expansion of the cell wall during mating projection growth requires constant assembly of new cell wall material in the expanding apical region (Fig 1B, 1C and 1F). Cell wall assembly occurs through synthesis of the primary component of the wall, 1,3-*β* glucan [44], by transmembrane 1,3-*β* glucan synthases Fks1/2, which localize at the apical, growing region of the mating projection [50, 51]. While only inactive Fks1/2 molecules, unable to synthesize glucans, are incorporated to the plasma membrane through exocytosis, Fks1/2 can be activated by Rho1 once at the plasma membrane [52] (Fig 1C and 1D). The activated form of Fks1/2 synthases extrudes 1,3-*β* glucan chains into the extracellular space, thereby assembling new cell wall onto the preexisting wall [4]. Accounting for these events, mass conservation of cell wall material yields

$$\partial_{t}\left(rh\right)+\partial_{s}\left(rhu\right)=\frac{rm_{m}k_{p}}{\rho_{w}}\;\rho_{A}\left(s,t\right)\;,$$ (3)

where *h*(*s*, *t*) is the cell wall thickness (Fig 1E), and *m*_*m*_ and *k*_*p*_ are the mass of a 1,3-*β* glucan monomer and the 1,3-*β* glucan assembly rate by Fks1/2 synthases, respectively. For simplicity, we assume that the assembly rate of new cell wall material is proportional to the local surface density *ρ*_A_ of active Fks1/2. Given that Fks1/2 synthases are constantly added and removed from the plasma membrane by exo- and endo-cytosis, it is important to consider their dynamics.

Inactive Fks1/2 are transported to the apical region of the mating projection by the cell’s exocytic machinery and incorporated to the plasma membrane via exocytosis [53] (Fig 1C). Once on the membrane, inactive Fks1/2 molecules, characterized by a local density *ρ*_*I*_, can be activated at a rate *k*_on_. Due to the relatively fast exo- and endocytosis Fks1/2 recycling (∼ 1s [54]) and very low diffusion constant *D* of proteins on yeast membranes (*D* ∼ 0.01 *μ*m^2^/*s* [55]), the diffusive movement of inactive Fks1/2 on the plasma membrane can be neglected. In the active state, Fks1/2 extrudes new 1,3-*β* glucan chains into the wall, which get assembled into the preexisting 1,3-*β* glucan network, effectively attaching active Fks1/2 to the wall and leading to a wall-driven convective movement of active Fks1/2 with velocity *u*. Finally, active Fks1/2 synthases become inactive at a rate *k*_off_ (Fig 1C). The dynamics for both inactive and active Fks1/2 states can be written in the curved geometry of the cell as

$$\begin{array}{c} \begin{array}{c} \partial_{t}\left(\rho_{I}r\right)=r\left[k_{\text{off}}\rho_{A}-k_{\text{on}}\rho_{I}\right]+r\left[k_{X}\rho_{0}-k_{D}\rho_{I}\right]\;,\\ \partial_{t}\left(\rho_{A}r\right)+\partial_{s}\left(\rho_{A}ru\right)=r\left[k_{\text{on}}\rho_{I}-k_{\text{off}}\rho_{A}\right]\;, \end{array} \end{array}$$ (4)

where *k*_X_ and *k*_D_ are the exocytosis and endocytosis rates, respectively. Experimental observations of the spatial distribution of both exocytic and endocytic events during mating projection growth indicate that these are maximal at the apex and decay away from it [54]. These localized exo- and endo-cytosis profiles are characterized by a decay length scale and can be written as $k_{X}\left(s,t\right)=k_{X}^{0}\;\text{exp}\left(-s^{2}/\lambda_{X}^{2}\right)$ and $k_{D}\left(s,t\right)=k_{D}^{0}\;\text{exp}\left(-s^{2}/\lambda_{D}^{2}\right)$, where $k_{X}^{0}$ and $k_{D}^{0}$ are the apical rates of exocytosis and endocytosis, respectively, and λ_*X*_ and λ_*D*_ are the length scales over which exocytosis and endocytosis decay, respectively. Given that the enzymes that locally degrade the cell wall and control its mechanical properties are secreted through exocytosis [56], we assume the length scale of viscosity variation to be set by the exocytosis length scale and write the viscosity profile as $\mu \left(s\right)=\mu_{0}\;\text{exp}\left(s^{2}/\lambda_{X}^{2}\right)$.

In order to simultaneously solve for the mechanics of cell wall expansion and the dynamics of cell wall assembly described above, it is necessary to specify the activation and inactivation rates of membrane-localized Fks1/2 cell wall synthases, *k*_on_ and *k*_off_, respectively. Inactivation of active, membrane-localized Fks1/2 synthases has been largely unexplored and assumed here to occur at a constant rate. The activation of inactive Fks1/2 is mediated by the GTPase Rho1 through the CWI pathway [21, 29, 33] (Fig 1D), providing a direct coupling between the local mechanical state of the wall and the local cell wall synthesis machinery via the Fks1/2 activation rate *k*_on_ (Fig 1D). To account for this coupling, we write the Fks1/2 activation rate *k*_*on*_ as dependent on the cell wall mechanical state, namely

$$k_{\text{on}}=\Gamma \;\left[{\dot{\epsilon }}_{s}+{\dot{\epsilon }}_{\phi }\right]\;,$$ (5)

where we assumed the stress sensors to perceive the expansion (strain) rate in the wall, rather than strain or stress. Indeed, activation of cell wall synthases should not occur in the absence of cell wall expansion, as it could otherwise lead to uncontrolled cell wall thickening. Eq 5 constitutes a direct *mechanical feedback of cell wall mechanics on cell wall assembly*, with the dimensionless parameter Γ establishing the strength of the mechanical feedback: large values of Γ indicate that low levels of cell wall expansion trigger large activation of Fks1/2 synthases, and vice versa.

Combining Eqs 1–5 and the profiles of exocytosis, endocytosis and wall viscosity described above, we solve the coupled dynamics of cell wall mechanical expansion and assembly. Normalizing all variables, we find 5 dimensionless parameters that control the dynamical regimes of the system (Table 1), namely $k_{\text{off}}/k_{X}^{0}$, $k_{D}^{0}/k_{X}^{0}$, λ_*D*_/λ_*X*_, Γ, and the ratio (*Pρ*_w_λ_X_)/(12*μ*_0_*m*_w_*ρ*_0_*k*_p_), which corresponds to the ratio λ_X_/λ_m_ of the exocytosis length scale λ_X_ and a length scale λ_m_ ≡ 12*μ*_0_ *m*_w_ *ρ*_0_ *k*_p_/*Pρ*_w_ set by the expansion mechanics of the cell wall. The parameters $k_{\text{off}}/k_{X}^{0}$, $k_{D}^{0}/k_{X}^{0}$, λ_*D*_/λ_*X*_ have either been measured or estimated and we use below their known values [54, 57] (Table B in S1 File); variations in these parameters do not qualitatively affect our results (S1 File). In contrast, the feedback strength Γ and the ratio (*Pρ*_w_λ_X_)/(12*μ*_0_*m*_w_*ρ*_0_*k*_p_) are unknown and we explore below the dynamical regimes of the system when these parameters are varied.

**Table 1:** System physical parameters and relevant dimensionless parameters.

Dimensionless parameters			
$\frac{P\rho_{w}\lambda_{X}}{12\;\mu_{0}m_{w}\rho_{0}k_{p}}$ Γ $\frac{k_{\text{off}}}{k_{X}^{0}}$ $\frac{k_{D}^{0}}{k_{X}^{0}}$ $\frac{\lambda_{D}}{\lambda_{X}}$			
Physical/Chemical parameters			
Parameter	Description	Parameter	Description
*P*	Turgor pressure of budding yeast	λ_*X*_	Exocytosis length-scale
*ρ*_*w*_	Density of 1,3-*β* glucans in cell wall	λ_*D*_	Endocytosis length-scale
*μ*_0_	Apical viscosity of cell wall	$k_{X}^{0}$	Apical rate of exocytosis
*m*_*w*_	Mass of 1,3-*β* glucan monomer	$k_{D}^{0}$	Apical rate of endocytosis
*ρ*_0_	Density of Fks1/2 enzymes in vesicle	*k*_off_	Inactivation rate of Fks1/2
*k*_*p*_	Extrusion rate of 1,3-*β* glucan monomers		

### Stability of mating projection growth

In the absence of mechanical feedback (Γ = 0) and only active Fks1/2 (see S1 File for details) mating projection growth is unstable for any value of the parameters. We find that in the absence of mechanical feedback the cell wall either progressively thins, eventually leading to either cell wall piercing, or thickens, leading to unbounded cell wall growth, depending on parameter values (S1 File). This instability arises from the lack of coordination between cell wall expansion and assembly: changes in cell wall expansion cannot be balanced by cell wall assembly unless the processes building the cell wall have information about how cell wall expansion is changing on the cell’s surface.

**Fig 2:**
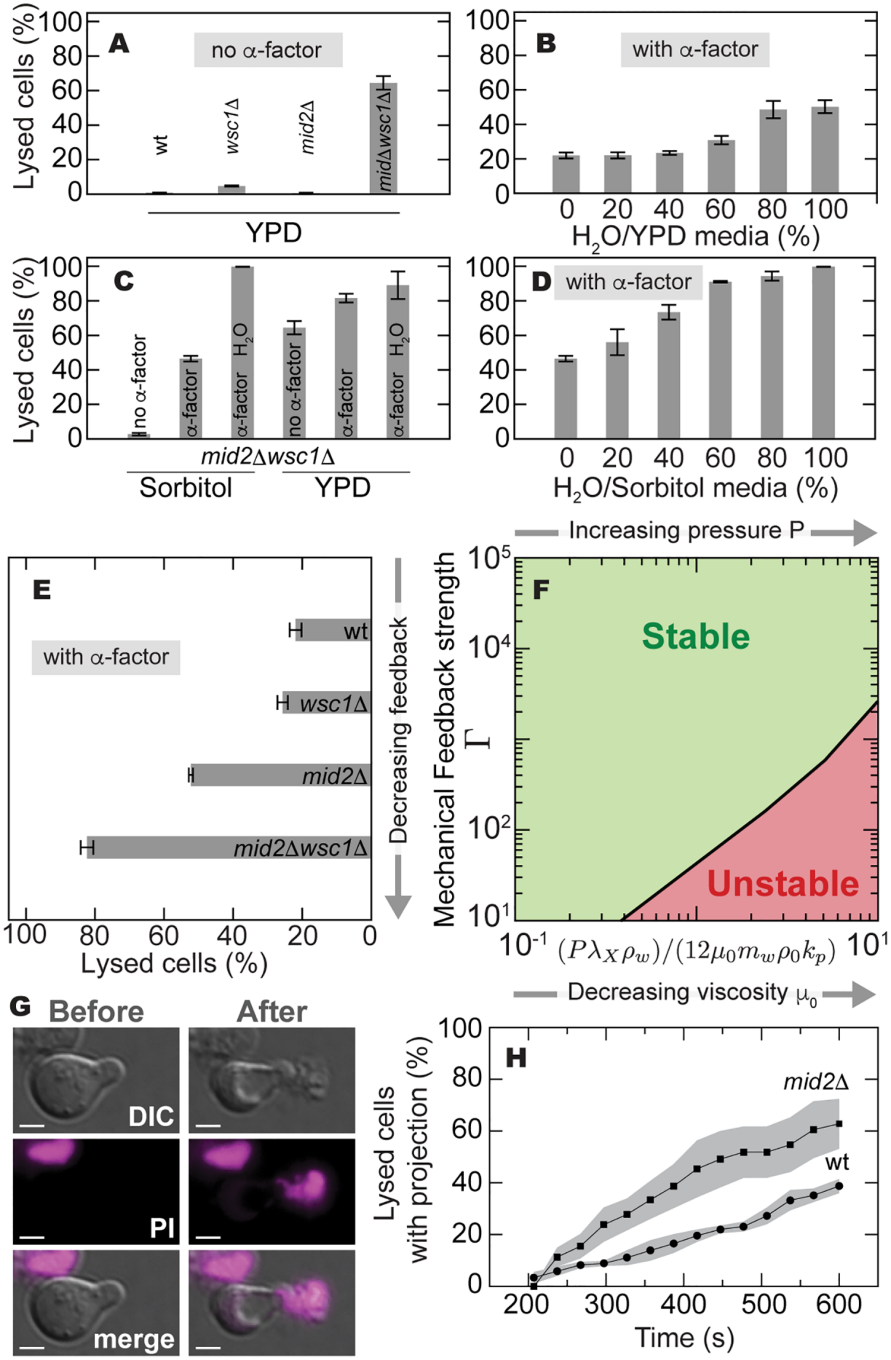
Effect of mechanical feedback strength and turgor pressure on cell viability. The strength of mechanical feedback, Γ, is experimentally varied by deleting *MID2* and *WSC1*. The dimensionless parameter (*Pρ*_w_λ_X_)/(12*μ*_0_*m*_w_*ρ*_0_*k*_p_) is varied by changing the osmolarity of the external medium through dilution of the yeast growth media, YPD, in deionized H_2_O, effectively increasing turgor pressure *P* in cells. Cell lysis was measured using the PI staining viability assay (Methods). (A) Percent of lysed cells in the absence of *α*-factor for WT, *mid2Δ* and *wsc1Δ* mutants, as well as the *mid2* *Δwsc1Δ* double mutant. (B) Percent of WT lysed cells when grown in the presence of *α*-factor in YPD medium with decreasing osmolarity. (C) Percent of *mid2Δ* *wsc1Δ* lysed cells when grown both in the presence and absence of *α*-factor in YPD, in osmotically supported conditions (YPD + 1M sorbitol), as well as in hypo-osmotic conditions (100% H_2_O). (D) Percent of *mid2Δ* *wsc1Δ* lysed cells when grown in the presence of *α*-factor and osmotically supported media (YPD + sorbitol), diluted for decreasing osmolarities. (E) Percent of lysed cells in *mid2Δ* and *wsc1Δ* mutants, as well as the *mid2Δwsc1Δ* double mutant, when grown in the presence of *α*-factor in YPD. (F) Theoretically predicted dynamical regimes for varying values of the mechanical feedback strength Γ and the ratio (*Pρ*_w_λ_X_)/(12*μ*_0_*m*_w_*ρ*_0_*k*_p_). Decreasing osmolarity experimentally, corresponds to increasing *P* and, therefore, moving along horizontal lines in the positive direction. Addition of zymolyase, a cell wall degrading enzyme, corresponds to decreasing the cell wall viscosity, moving also along horizontal lines in the positive direction. (G) Images (DIC, PI staining and merge) showing the moments before and after the piercing of the cell wall at the tip of a mating projection and subsequent cell lysis of a *mid2Δ* cell after the addition of zymolyase, for video see S1 Video. Scale bar, 2*μm*. (H) Temporal increase in the fraction of pierced mating projections for both *mid2Δ* (squares) and WT (circles) cells after addition of zymolyase.

In the presence of mechanical feedback (Γ > 0), numerical integration of Eqs 1–5 (Methods) shows that stable states of mating projection growth can be sustained for a large range of parameters (Fig 2F and S1 File). In this context, stable states refer to sustained steady state growth of the mating projection at constant velocity. For any given value of the ratio (*Pρ*_w_λ_X_)/(12*μ*_0_*m*_w_*ρ*_0_*k*_p_) there exists a critical value of the feedback strength Γ below which mating projection growth is unstable. Similarly, for every value of the feedback strength Γ, there is a maximal value of (*Pρ*_w_λ_X_)/(12*μ*_0_*m*_w_*ρ*_0_*k*_p_) above which mating projection growth becomes unstable. This instability is caused by the progressive thinning of the apical cell wall, eventually causing the piercing of the cell and leading to cell lysis. The bifurcation between stable and unstable states characterizes the transition between stably growing mating projections and a situation in which this stable growth cannot be sustained because of the progressive thinning of the cell wall and its eventual piercing. This instability threshold (bifurcation) is equivalent to the existence of a maximal turgor pressure (or a minimal viscosity), above (below) which the cell wall progressively thins and eventually pierces at the tip of the projection, leading to cell lysis. The predicted increase of the maximal turgor pressure or decrease in the minimal wall viscosity for increasing feedback strength Γ indicates that cells with compromised mechanical feedback should be more sensitive to both an increase in turgor pressure or a decrease in wall viscosity than WT cells.

In order to experimentally explore the predicted dynamical regimes (Fig 2F), we systematically varied the mechanical feedback strength, as well as the turgor pressure *P* and cell wall viscosity *μ*_0_. In contrast to previous works, here we examine all three perturbations in the context of the stability of pheromone-induced projection growth. We first varied the feedback strength Γ by compromising the ability of the cell to sense the mechanical state of the wall. To this end, we genetically deleted the two primary cell wall stress sensors present during mating projection growth, namely Wsc1 and Mid2 [33] (Fig 1D), and measured the resulting cell lysis (Methods). Only in the presence of *α*-factor and mating projection growth, did the deletion of either of the two sensors (Mid2, Wsc1) lead to increased levels of cell lysis compared to WT (Fig 2A and 2E), as predicted theoretically (Fig 2F), indicating that the ability to sense the mechanical state of the wall is essential during growth. Moreover, the double mutant *mid2Δ*wsc1*Δ* exhibited the highest level of cell lysis in *α*-factor and, even when osmotically supported by 1M sorbitol, showed a substantial increase in lysis after the addition of *α*-factor (Fig 2A, 2C and 2E). These observations show that the double mutant has an enhanced sensitivity to the addition of mating pheromone, in agreement with previous results obtained during vegetative growth [40]. To explore how changes in the parameter (*Pρ*_w_λ_X_)/(12*μ*_0_*m*_w_*ρ*_0_*k*_p_) affected cell viability (Fig 2F), we independently changed the turgor pressure *P* and the cell wall viscosity *μ*_0_. To increase the cell’s turgor pressure *P*, we progressively decreased the osmolarity of the external medium (Methods). We observed a monotonic increase in lysed cells for both WT and *mid2Δ*wsc1*Δ* cells as media osmolarity was decreased in the presence of *α*-factor (Fig 2B and 2D), consistent with the theoretically predicted effect of increased turgor pressure *P* (Fig 2F). Finally, in order to decrease the cell wall viscosity *μ*_0_, thereby increasing the value of the parameter (*Pρ*_w_λ_X_)/(12*μ*_0_*m*_w_*ρ*_0_*k*_p_) (Fig 2F), we added zymolyase to the culture media (Methods). Zymolyase enzymatic activity degrades 1,3-*β* glucans in the cell wall, effectively lowering the cell wall viscosity. Addition of zymolyase led to the piercing of the cell wall typically at the tip of the mating projection (Fig 2G and Supplementary Video), as expected theoretically (Fig 2F). Since zymolyase will continuously degrade the cell wall, leading to the eventual piercing and lysis of all cells, we studied the temporal increase in pierced cells. Our results indicate that *mid2Δ* cells with reduced mechanical feedback pierced faster than WT cells when grown at the same zymolyase concentration (Fig 2H), as theoretically expected (Fig 2F). Overall, our experimental results are in agreement with our theoretical predictions (Fig 2F) and are consistent with the CWI pathway providing the necessary mechanical feedback to coordinate cell wall expansion and assembly.

### Characteristics of stably growing mating projections

**Fig 3:**
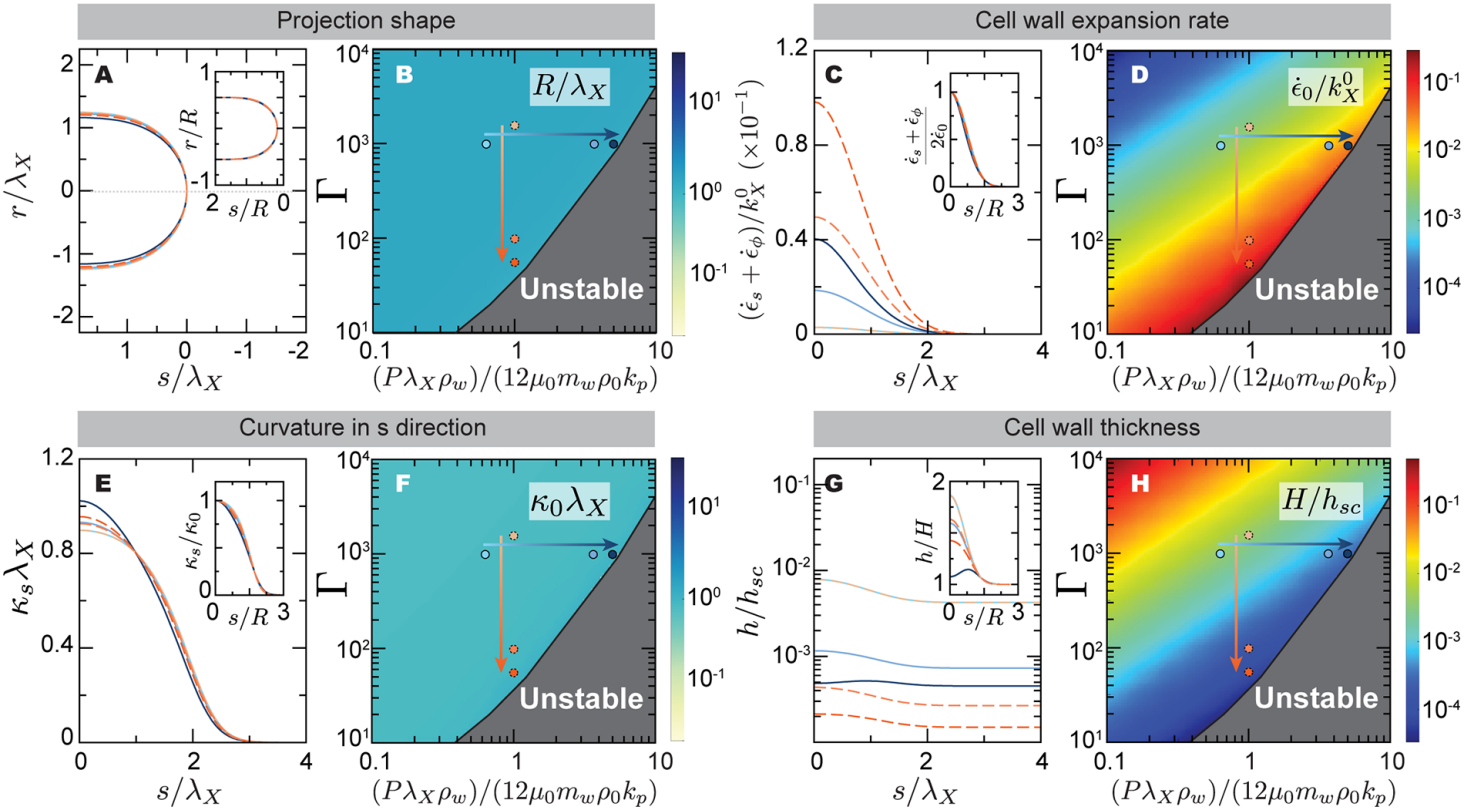
Steady-state stable solutions for mating projection growth: Projection shape and cell wall expansion. (A,C,E,G) Mating projection shape (A), as well as the spatial profiles of the cell wall expansion rate ${\dot{\epsilon }}_{s}+{\dot{\epsilon }}_{\phi }$ (C), curvature *κ*_*s*_ (E) and cell wall thickness *h* (G), for different values of the mechanical feedback strength Γ and the ratio λ_X_/λ_m_ = (*Pρ*_w_λ_X_)/(12*μ*_0_*m*_w_*ρ*_0_*k*_p_). All insets show a different scaling of each magnitude, with the arclength normalized by the projection radius *R* and each quantity normalized by its value at the projection tip (*s* = 0), with the exception of the wall thickness *h*(*s*) and the shape *r*(*s*), which are normalized by the limiting values far away from the apical region, *H* and *R* respectively. The color code indicates the different parameter values, shown as dots of the same color in the parameter space right to each panel. Increasing orange and blue tones of the dots corresponds to decreasing Γ and increasing (*Pρ*_w_λ_X_)/(12*μ*_0_*m*_w_*ρ*_0_*k*_p_), respectively (arrows in Fig 2E). (B,D,F,H) The variation of the apical value of each magnitude, namely ${\dot{\epsilon }}_{0}\equiv {\dot{\epsilon }}_{s}\left(s=0\right)={\dot{\epsilon }}_{\phi }\left(s=0\right)$ (D) and *κ*_0_ ≡ *κ*_*s*_(*s* = 0) (F), is shown for the different values of the parameters for which stable states exist. The variation of the projection radius and wall thickness away from the apical region, *R* (B) and *H* (H) respectively, are shown as a function of the parameters as well.

Stable, steady-state solutions for mating projection growth show that the shape of the mating projection is largely insensitive to variations in the feedback strength Γ and the ratio (*Pρ*_w_λ_X_)/(12*μ*_0_*m*_w_*ρ*_0_*k*_p_) (Fig 3A, 3B, 3E and 3F). The size (radius) *R* of the mating projection increases linearly with the size of the exocytosis region λ_*X*_, but it is independent from the feedback strength Γ (Fig 3B). Beyond projection shape and size, the cell wall expansion rate, ${\dot{\epsilon }}_{s}+{\dot{\epsilon }}_{\phi }$, is always maximal at the projection apex (*s* = 0) and decreases away from it (Fig 3C), eventually vanishing as no wall expansion occurs far away from the growing apical region. The cell wall expansion rate at the projection apex, $\left({\dot{\epsilon }}_{s}+{\dot{\epsilon }}_{\phi }\right){|}_{s=0}$, increases with increasing turgor pressure (or (*Pρ*_w_λ_X_)/(12*μ*_0_*m*_w_*ρ*_0_*k*_p_) equivalently) and with decreasing mechanical feedback strength (Fig 3C and 3D). In contrast, the apical cell wall thickness displays the opposite behavior (Fig 3G and 3H), decreasing for increasing *P* or decreasing Γ. These results indicate that cells closer to the instability threshold display stronger apical cell wall expansion rates and thinner cell wall (Figs 2E and 3D and 3H), suggesting the strong cell wall expansion and thinning at the apex as the cause of the loss in cell wall mechanical stability.

**Fig 4:**
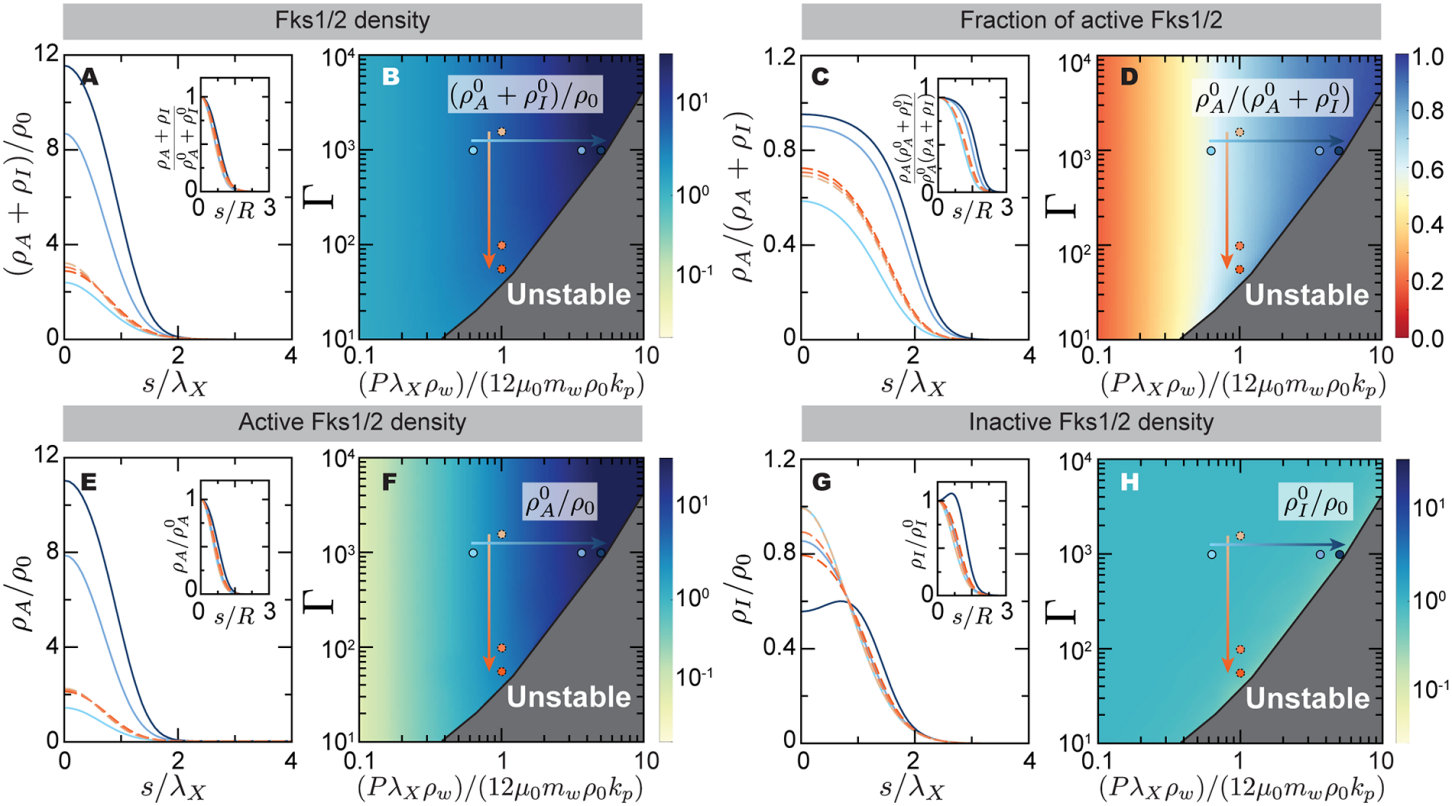
Steady-state stable solutions for mating projection growth: Cell wall assembly via Fks1/2. Cell wall assembly via Fks1/2. (A,C,E,G) Total Fks1/2 density *ρ*_*A*_ + *ρ*_*I*_ (A), fraction of active Fks1/2, *ρ*_*A*_/(*ρ*_*A*_ + *ρ*_*I*_) (C), active Fks1/2 density (E) and inactive Fks1/2 density (E), for different values of the mechanical feedback strength Γ and the ratio λ_X_/λ_m_ = (*Pρ*_w_λ_X_)/(12*μ*_0_*m*_w_*ρ*_0_*k*_p_). All insets show a different scaling of each magnitude, with the arclength normalized by the projection radius *R* and each quantity normalized by its value at the projection tip (*s* = 0). The color code indicates the different parameter values, shown as dots of the same color in the parameter space right to each panel. Increasing orange and blue tones of the dots corresponds to decreasing Γ and increasing (*Pρ*_w_λ_X_)/(12*μ*_0_*m*_w_*ρ*_0_*k*_p_), respectively (arrows in Fig 2E). (B,D,F,H) The variation of the apical value of each magnitude, namely $\left(\rho_{A}^{0}+\rho_{I}^{0}\right)/\rho_{0}$ (B), $\rho_{A}^{0}/\left(\rho_{A}^{0}+\rho_{I}^{0}\right)$ (D), $\rho_{A}^{0}/\rho_{0}$ (F) and $\rho_{I}^{0}/\rho_{0}$ (H), is shown for the different values of the parameters for which stable states exist.

Regarding cell wall assembly during stable, steady-state projection growth, our theoretical results indicate maximal cell wall assembly at the expanding apical region. Both the total surface density of Fks1/2 synthases, *ρ*_*A*_ + *ρ*_*I*_, and the surface density of only active Fks1/2 synthases, *ρ*_*A*_, are maximal at the apex and decrease away from it until they vanish (Fig 4A, 4B, 4E and 4F), as expected from the apically-localized exo- and endo-cytosis profiles. The apical value of the total (or only active) Fks1/2 surface density, namely $\rho_{A}^{0}+\rho_{I}^{0}$ (or $\rho_{A}^{0}$), can be either smaller or larger than the surface density *ρ*_0_ of Fks1/2 synthases secreted by exocytic vesicles (Fig 4A, 4B, 4E and 4F). The reason why the total Fks1/2 surface density $\rho_{A}^{0}+\rho_{I}^{0}$ can be larger than *ρ*_0_ at the apex is that active Fks1/2 is secreting 1,3-*β* glucans into the cell wall, a process that effectively anchors them to the wall, holding secreted Fks1/2 synthases to the tip region and increasing its concentration there. Beyond Fks1/2, anchoring transmembrane proteins to the cell wall can potentially be used as a mechanism to locally increase the protein concentration on the membrane to levels well-beyond secretion levels. The fraction of active Fks1/2, *ρ*_*A*_/(*ρ*_*A*_ + *ρ*_*I*_), is also maximal at the apex and decreases away from it (Fig 4C and 4D). This is because of mechanical feedback, which induces more Fks1/2 activation at the apex following the larger cell wall expansion rate in this region (Fig 3C). Finally, the surface concentration of inactive Fks1/2 also decreases away from the expanding tip because of tip-localized exo- and endo-cytosis (Fig 4G and 4H). Non-monotonic profiles of inactive Fks1/2 occur because high cell wall expansion rates at the tip lead to more Fks1/2 activation, leaving less inactive Fks1/2 molecules in this region. Altogether, these results indicate that at the instability threshold, the apical cell wall expansion rate becomes too large to be balanced by cell wall assembly, leading to the progressive thinning of the cell wall and cell lysis.

**Fig 5:**
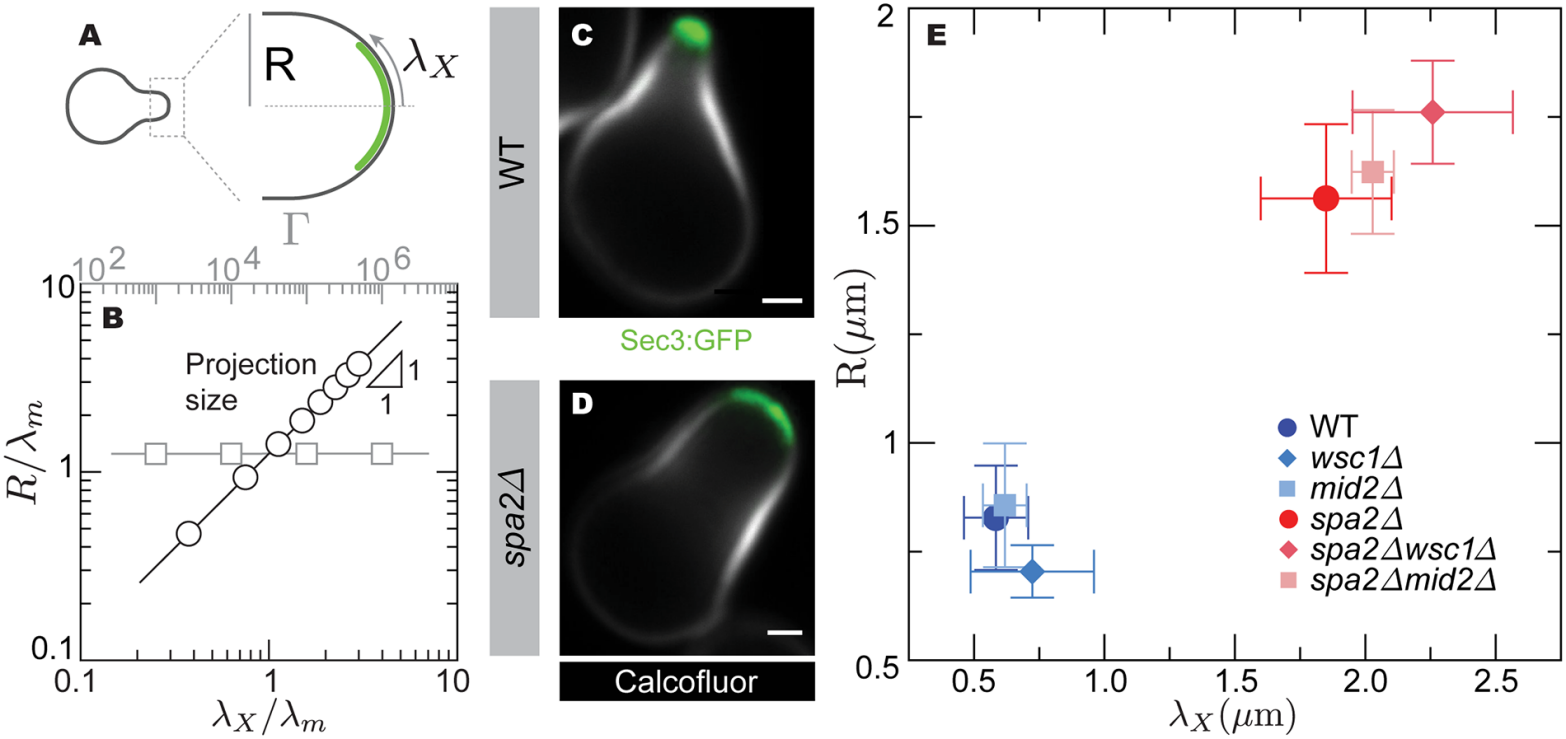
Control of mating projection size. (A) Diagram of a growing mating projection showing the mating projection radius *R* and length scale of the secretion region (green), λ_X_. (B) Theoretically predicted dependence of the projection radius *R* with the length scale of the secretion region, λ_X_, and the strength of mechanical feedback, Γ. (C-D) Confocal images of WT (C) and *spa2Δ* (D) mutant cells growing mating projections. The cell wall is labeled with calcofluor (white) and the exocytosis profile is defined by Sec3-GFP (green). Scale bar, 1 *μ*m. (E) Measured average cell radius, *R*, and exocytosis length scale, λ_X_, for *mid2Δ* and *wsc1Δ* mutants in both WT and *spa2Δ* backgrounds (*mid2Δ*, N = 6; *wsc1Δ*, N = 9; *spa2Δ* *mid2Δ*, N = 7; *spa2Δ* *wsc1Δ*, N = 6), as well as for WT (N = 7) and *spa2Δ* (N = 6) cells. Mean and standard deviation are shown.

The theoretical results above predict that both the geometry and size of the growing mating projection are independent from the mechanical feedback strength Γ, and that the projection radius increases with the size of the exocytosis region (Figs 5A and 5B and 3A and 3B). To experimentally explore how exocytosis and the mechanical feedback strength affect the mating projection size *R* (Fig 5A), we employed a deletion mutant for Spa2, a scaffold protein that localizes Bni1 and is recruited by Cdc42 [23], which displays a very wide mating projection compared to WT (Fig 5C and 5D). We visualized the exocytosis region in both WT and *spa2Δ* cells by expressing GFP-tagged Sec3, a component of the exocyst that marks exocytic sites [58]. The exocytosis length scale λ_X_ (Fig 5A), which we measured directly from confocal images (Fig 5C and 5D and Methods), is considerably larger in *spa2Δ* mutant cells than in WT cells (Fig 5E), indicating that a larger mating projection radius *R* is associated with a larger size of the exocytosis region. In contrast, the size *R* of the mating projection was not observed to vary with changes in the strength of the mechanical feedback Γ (Fig 5E), as shown by deleting Mid2 or Wsc1 in both WT and *spa2Δ* backgrounds, while simultaneously measuring the size of the mating projection *R* and the length of the exocytosis region using Sec3-GFP. While deletion of Wsc1 and Mid2 strongly affects mating projection stability (Fig 2E), our measurements show that it does not affect the size of the mating projection (Fig 5E). These results indicate that the mechanical feedback is essential to sustain stable mating projection growth, but it does not affect mating projection size, which is controlled by the exocytosis profile, as predicted theoretically (Fig 5B).

## Discussion

In this work, we studied both theoretically and experimentally how the mechanics of cell wall expansion and the molecular processes assembling the cell wall are coordinated during cell morphogenesis, using budding yeast mating projection growth as a model system. We first derived a theoretical description of mating projection growth that couples, through a mechanical feedback encoded in the CWI pathway, the cell wall expansion and geometry to the molecular processes building the cell wall. The theoretical predictions were tested experimentally through genetic deletions affecting the feedback strength and also through mechanical perturbations (hyposmotic shocks and cell wall degradation). Our theoretical predictions are in good agreement with the experimental results and indicate that the existence of mechanical feedback is essential to guarantee stability during cell wall remodeling and cell morphogenesis.

This theoretical description of mating projection growth connects the mechanics of the cell wall to the molecular events in charge of sensing its mechanical state and controlling its assembly via well-established signaling pathways (CWI pathway), thus providing specific predictions on how mutations can affect cell morphogenesis. Various previous models accounted for both the mechanics and assembly (remodeling) of the cell wall [16–19], as we have done above, but did not account for a connection to known molecular feedback control (CWI pathway) coupling wall mechanics and assembly. These models consider the cell wall to be either a elastic material undergoing remodeling [17, 18] or an elastoplastic material [16], as opposed to our description of the cell wall as a viscous fluid, which has also been considered before [19]. Importantly, at long timescales over which cell growth and cell wall remodeling occur, assuming the cell wall to be a viscous fluid, a remodeled elastic material or an elastoplastic material is largely equivalent because all of them properly account for the observed irreversible expansion (flow) of the cell wall at long timescales [16]. While previous descriptions assumed that irreversible cell wall expansion only occurs when new cell wall material is inserted into the pre-existing wall [17, 18], we allowed the possibility of cell wall expansion even in the absence of cell wall assembly because the cell wall can be fluidized by the action of wall degrading enzymes secreted via exocytosis. Indeed, addition of zymolyase leads to cell wall piercing for cells with intact cell wall assembly (Fig 2H). Such cell wall degrading enzymes are known to play an important role in cell wall remodeling [29, 59] and the establishment of inhomogeneous cell wall material properties in several organisms [47, 60], including budding yeast [16]. Since these enzymes are secreted via exocytosis, we assumed the length scale of viscosity variation away from the apex to be the same as the exocytosis region. Finally, the combination of the observed inhomogeneous stiffness of the cell wall during mating projection growth [16] (measured at short timescales; seconds) and cell wall remodeling can be theoretically described as an effective inhomogeneous viscosity at timescales longer than cell wall remodeling, as we assumed in our description above and also done previously for other systems [19].

We theoretically find that in the absence of any mechanical feedback relaying information about the mechanical state of the cell wall to the intracellular processes building it, cell wall expansion is unstable, leading to cell lysis. Previous works have shown that the cell wall is prone to piercing in cell if the CWI is compromised [29], and our experimental data indicates that degradation of the cell wall by zymolyase (effectively lowering the cell wall viscosity in our description) also leads to cell wall piercing (Fig 2H). Since cell wall piercing involves changes in cell wall thickness, our theoretical description accounts for the dynamics of cell wall thickness from first principles (mass conservation). This is in contrast to previous models that also consider cell wall mechanics and assembly, which assume the cell wall thickness to be constant, fixed by an unknown mechanism [16–18]. Considering a variable cell wall thickness was done before [19], but the cell wall mechanics and assembly were considered independently (no mechanical feedback) and the dynamics of cell growth was not studied. We theoretically show that accounting for the simplest mechanical feedback encoded in the CWI pathway, which directly couples cell wall expansion and assembly via direct activation of Fks1/2 synthases, stabilizes cell wall expansion for a wide range of parameters. The agreement between our theoretical predictions and experimental results suggests that the specific mechanical feedback studied herein, with cell wall stress sensors Wsc1 and Mid2 locally sensing cell wall expansion and directly activating Fks1/2 cell wall synthases, can stabilize cell wall remodeling during mating projection growth by itself. Such mechanical feedback ensures that in regions where the cell wall expands the fastest (at the projection apex) and could potentially rupture via thinning, local activation of cell wall synthases increases assembly of cell wall material, preventing cell wall rupture and stabilizing mating projection growth. However, our work does not rule out that other mechanical feedbacks encoded in the CWI pathway could also play a role in the stabilization of projection growth. It also is likely that other stress sensors [28, 29], expressed during different cell wall remodeling events in budding yeast, coordinate cell wall expansion and assembly in other morphogenetic processes. While our experimental observations qualitatively agree with our theoretical predictions regarding the existence of an instability associated to the thinning of the cell wall and then need of a mechanical feedback to coordinate cell wall extension and assembly, further experiments will be needed to fully confirm this scenario.

Beyond budding yeast, many other organisms, including other fungi, plants and bacteria, have walled cells that are constantly remodeled [5, 9, 61, 62]. The molecular control of cell wall remodeling and morphogenesis differs across species, and it is therefore likely that different mechanisms encode mechanical feedback in other species. Indeed, previous observations have hinted at the existence of mechanical feedback [63], but the feedback mechanisms remain elusive. The mechanical feedback described herein, or different feedback mechanisms to be discovered, may also play an important role in the coordination of cell polarity and morphogenesis in both animal and walled cells [63–66].

While essential to ensuring stability during cell wall expansion, our results show that the strength of mechanical feedback does not affect mating projection shape or size (Figs 3 and 5). The observed decoupling in the control of cell geometry and growth stability reported here may allow cells to maintain a functional shape under different growth conditions. In addition, we find that projection size is controlled by the spatial extent of exocytosis. This is in agreement with recent observations in fission yeast indicating that the size of the apical growth domain correlates best with the size of the apical exocytosis region [17], and also with theoretical models of fission yeast that predict the radius of the cell to be determined by the size of the apical cell wall assembly region [18].

More generally, the need to coordinate growth processes and mechanics during morphogenesis is important for individual cells, but also for tissues and organs. Identifying the molecular mechanisms enabling this coordination at different scales and in different organisms will substantially contribute to our understanding of morphogenetic processes.

## Supporting information

S1 File Supporting information file. (PDF)

S1 Video Video of cell piercing at tip of mating projection. A mid2*Δ* cell growing a mating projection is shown after the addition of zymolyase. The cell pierces at the tip as theoretically predicted. From left to right, DIC image, PI staining, and merge.
(AVI)
