# Supplementary material for: Mechanical feedback coordinates cell wall expansion and assembly in yeast mating morphogenesis
Source: PLoS Comput Biol. 2018 Jan 18;14(1):e1005940. doi: 10.1371/journal.pcbi.1005940 (PMC5790295; doi:10.1371/journal.pcbi.1005940)
Supplement: S1 File — (PDF) [file pcbi.1005940.s001.pdf]

Supporting Information

Mechanical feedback coordinates cell wall expansion and assembly in yeast mating morphogenesis

Samhita Banavar, Carlos Gomez, Michael Trogdon,

Linda Petzold, Tau-Mu Yi & Otger Campàs

Mating projection growth is unstable in the absence of mechanical feedback

In the absence of mechanical feedback ($\Gamma = 0$), the equations in the main text cannot lead to projection growth because Fks1/2 synthases cannot be activated and, consequently, there is no cell wall assembly at all. To study the system in the absence of mechanical feedback ($\Gamma = 0$), but in the presence of cell wall assembly, we only consider an active Fks1/2 state, with Fks1/2 molecules incorporated to the plasma membrane via exocytosis and removed from it via endocytosis. As Fks1/2 molecules are always active in this limiting case, they directly contribute to the assembly of the cell wall and their dynamics is fully decoupled from the cell wall mechanics, namely

$$\partial_t(\rho_A r) + \partial_s(\rho_A r u) = r[k_X \rho_0 - k_D \rho_A]\,. \tag{S1}$$

Eqs. 1-3 in the main text and Eq. S1, together with the profiles of exocytosis, endocytosis and wall viscosity described in the main text, describe the coupled dynamics of cell wall mechanical expansion and assembly in the absence of mechanical feedback. Normalizing all variables, we obtain the relevant dimensionless parameters, namely $k^0_{\rm D}/k^0_{\rm X}$, $\lambda_{\rm X}/\lambda_{\rm D}$, and the ratio $(P\rho_{\rm w}\lambda_{\rm X})/(12\,\mu_0 m_{\rm w}\rho_0 k_{\rm p})$, which corresponds to the ratio $\lambda_{\rm X}/\lambda_{\rm m}$ of the exocytosis length scale $\lambda_{\rm X}$ and a length scale $\lambda_{\rm m} \equiv 12\,\mu_0 m_{\rm w}\rho_0 k_{\rm p}/P\rho_{\rm w}$ set by the expansion mechanics of the cell wall.

We numerically integrate the dynamics of the system in the absence of feedback and find that no stable states exist for varying values of k^0_D/k^0_X and λ_X/λ_m and fixed $\lambda_X/\lambda_D = 0.5$ or for λ_X/λ_D and λ_X/λ_m fixed $k^0_D/k^0_X = 1.0$ (Fig. S1A,B). No stable states were found over a two order of magnitude variation of the parameters around their unit values. The instability is caused by either the progressive thinning or thickening of the cell wall, depending on the region of the parameters space (Fig. S1A,B). Cell wall thinning eventually leads to the piercing of the cell wall at the apex ($h(s = 0, t_p) = 0$) at a finite time t_p (Fig. S1C). In other regions of the parameters space, the apical cell wall progressively thickens without bound (Fig. S1D). The presence of a mechanical feedback is therefore crucial for the stable growth of the mating projection. In the absence of a mechanical feedback there is a lack of coordination between cell wall expansion and wall assembly, making it impossible for the cell wall synthesis machinery (Fks1/2) to appropriately balance cell wall expansion because it does not have information about its mechanical state.

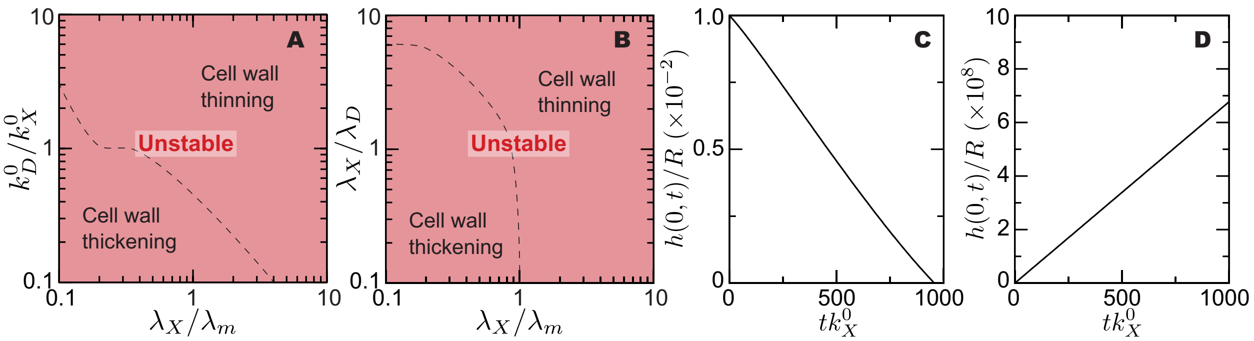

Fig. S1. Mating projection growth is always unstable in the absence of mechanical feedback. (A) No stable solutions exist as k^0_D/k^0_X and λ_X/λ_m are varied and $\lambda_X/\lambda_D = 0.5$. (B) No stable solutions exist as λ_X/λ_D and λ_X/λ_m are varied and $k^0_D/k^0_X = 1.0$. (C) Progressive thinning of the apical cell wall ($h(s = 0, t)$), eventually leading to the piercing of the cell wall at the apex. (D) Progressive, unbounded thickening of the apical cell wall.

Dynamical regimes of the system

The dynamical regimes shown in the main text were obtained by varying the mechanical feedback strength Γ and the ratio $(P\rho_{\mathrm{w}}\lambda_{\mathrm{X}})/(12\,\mu_0 m_{\mathrm{w}}\rho_0 k_{\mathrm{p}}) = \lambda_{\mathrm{X}}/\lambda_{\mathrm{m}}$, while keeping all other parameters fixed at values consistent with previous experimental observations in mating projection growth ($\lambda_X/\lambda_D = 0.5$, $k_D^0/k_X^0 = 2.0$, and $k_{\mathrm{off}}/k_X^0 = 1.0$). Here we vary the parameters held constant in the main text within a reasonable range and determine the dynamical regimes. Since our theoretical predictions and experimental results indicate that the instability occurs as the pressure is increased or the mechanical feedback decreased, we explore if this behavior is robust as we vary all other parameters. Varying the parameters k_D^0/k_X^0 and k_{off}/k_X^0 by two orders of magnitude (from 0.10 to 10) does not change our qualitative results (Fig. S2): there is transition between stable and unstable states, and the critical value of the mechanical feedback strength increases with $\lambda_{\mathrm{X}}/\lambda_{\mathrm{m}}$. Varying λ_X/λ_D leads also to the same qualitative results (Fig. S3).

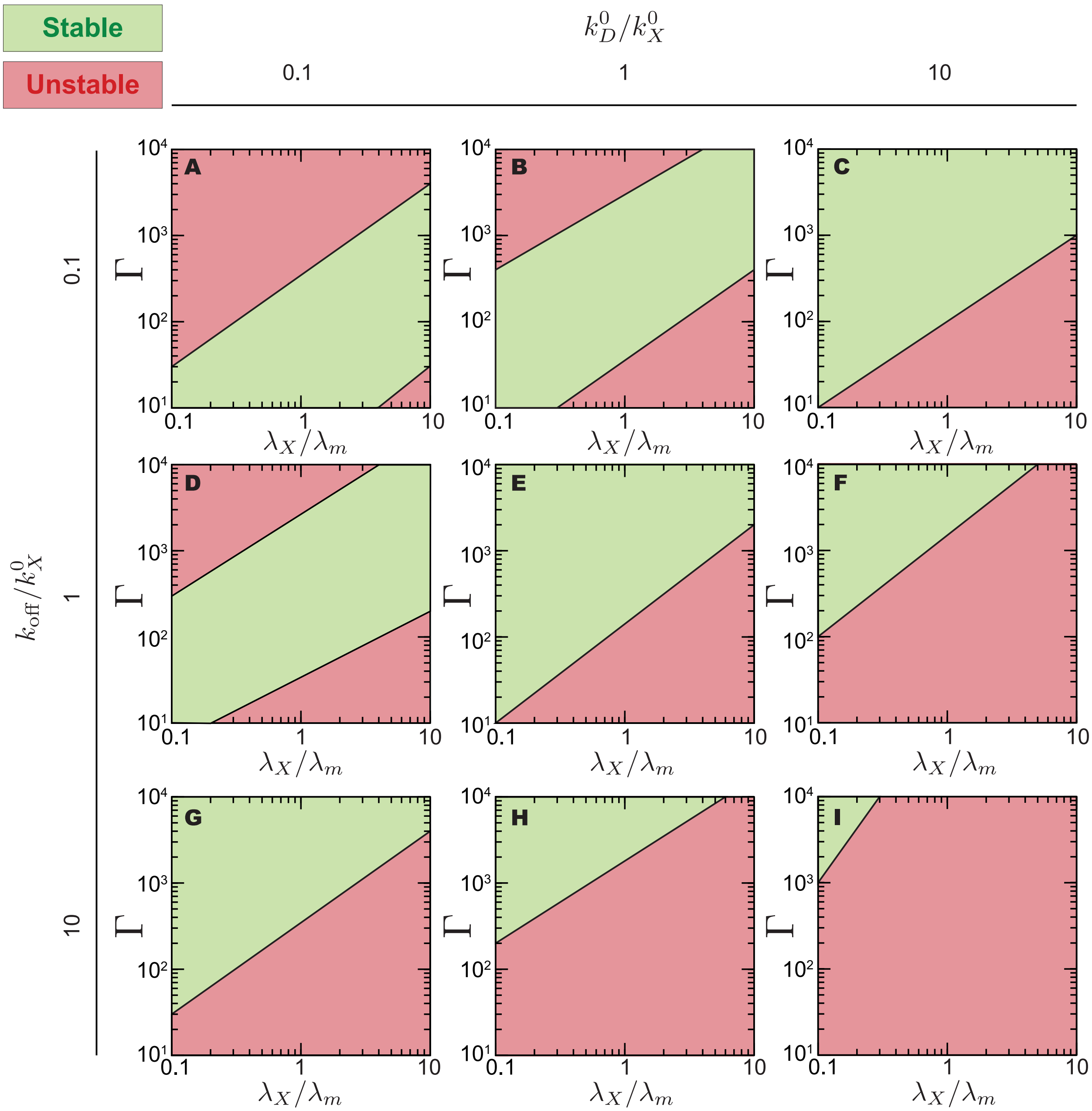

Fig. S2. Dynamical regimes Parameter space spanned by Γ and λ_X/λ_m (A-I) for different values of the parameters k_D^0/k_X^0 and k_{off}/k_X^0. The transition from unstable growth (red) to stable growth (green) exists in all cases.

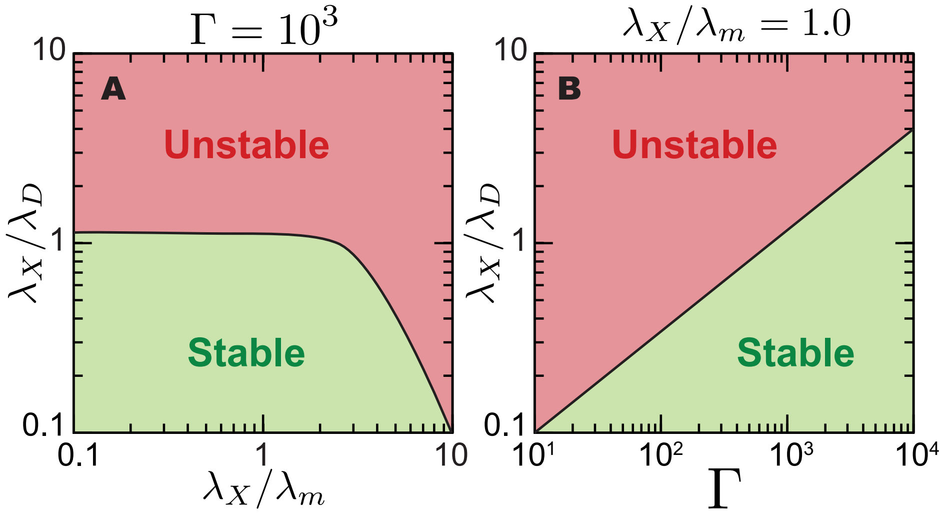

Fig. S3. Dynamical regimes as the ratio of lengthscales of exocytosis and endocytosis is varied Transition from unstable (red) growth to stable (green) growth exists in all cases.

Scaling relations and steady-state solutions

Analytical expressions can be obtained for the values of all variables at the apex (apical scales), but one. This is because the two principal directions defining the cell's surface become physically equivalent at the apex (as the apex is locally spherical) and, as a consequence, there are not enough boundary conditions at the apex to determine all apical scales. This can be seen from the analytical relations of the apical scales (Eq. S2), which are defined as: $\kappa_0 \equiv \kappa_s(s=0) = \kappa_\phi(s=0)$, $h_0 \equiv h(s=0)$, $\dot{\epsilon}_0 \equiv \dot{\epsilon}_s(s=0) = \dot{\epsilon}_\phi(s=0)$, $\rho_A^0 \equiv \rho_A(s=0)$ and $\rho_I^0 \equiv \rho_I(s=0)$.

$$\begin{array}{|l|}\hline h_0 = A\frac{k_p}{k_{off}}\frac{k_X^0}{k_D^0}\frac{m_w\rho_0}{\rho_w} \\ \hline \kappa_0\dot{\epsilon}_0 = \frac{1}{A}\frac{k_D^0}{k_X^0}\frac{k_{off}}{k_p}\frac{\rho_w}{m_w\rho_0}\frac{P}{12\mu_0} \\ \hline \rho_I^0 = \frac{k_X^0}{k_D^0}\rho_0 \\ \hline \kappa_0\rho_A^0 = \frac{P}{6\mu_0 k_p}\frac{\rho_w}{m_w\rho_0}\rho_0 \\ \hline \end{array} \tag{S2}$$

To fully determine all the scales, it is necessary to numerically integrate the equations describing the dynamics of the system and impose boundary conditions away from the apex. In this region, the projection radius reaches a constant distal value $R \equiv r(s \to \infty)$ and the cell wall thickness reaches a constant distal value $H = h(s \to \infty)$.

Integrating numerically the equations describing the dynamics of the system in the steady-state (see main text), we find the solutions for all the variables (see main text). We also obtain the values of all scales as the parameters Γ and the ratio $(P\rho_{\rm w}\lambda_{\rm X})/(12\,\mu_0 m_{\rm w}\rho_0 k_{\rm p}) = \lambda_{\rm X}/\lambda_{\rm m}$ are varied. The scaling relations defining the dependence of the scales on the parameters Γ and $\lambda_{\rm X}/\lambda_{\rm m}$ are shown in Fig. S4.

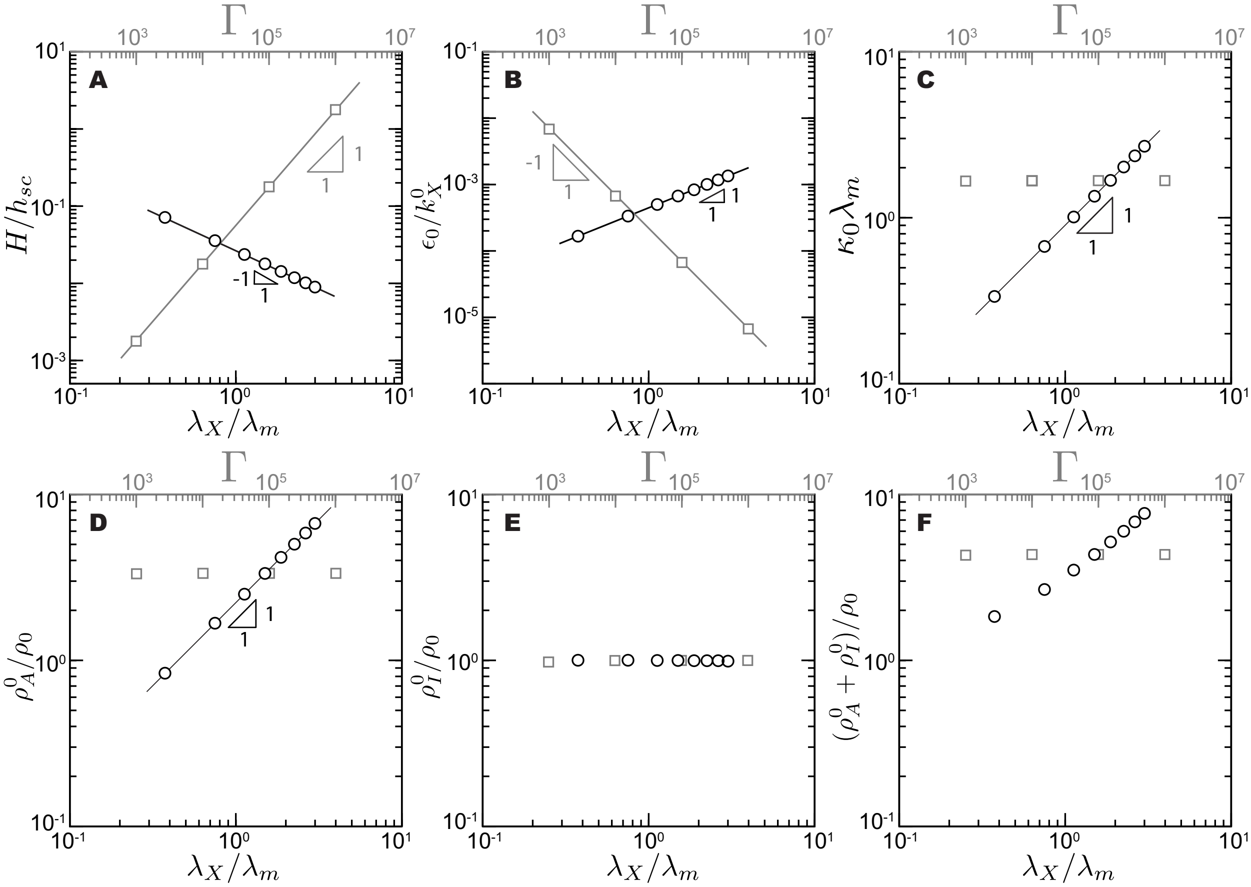

Fig. S4. Dependence of the apical and distal scales on Γ and λ_X/λ_m (A) Distal thickness, H, scaled with $h_{sc} = P\lambda_X/12\mu_0 k_X^0$, (B) apical strain rate, ϵ_0 scaled with k_X^0, (C) apical curvature, κ_0 scaled with λ_m, (D) apical density of active Fks1, ρ_A^0, scaled with ρ_0, (E) apical density of inactive Fks1, ρ_A^0, scaled with ρ_0, and (F) apical density of Fks1, $\rho_A^0 + \rho_I^0$, scaled with ρ_0. Exponents of power-law behaviors are indicated.

Table A:

**Strain**	**Genotype**	**Source**
RJD863	*MAT**a** can1-100 leu2-3-112 his3-11-15 trp1-1 ura3-1 ade2-1 bar1::hisG*	Ray Deshaies
CGY003	RJD863 *wsc1Δ::KAN*^{R}	This study
CGY004	RJD863 *mid2Δ::KAN*^{R}	This study
CGY005	RJD863 *wsc1Δ::HIS5 mid2Δ::KAN*^{R}	This study
CGY011	RJD863 *sec3Δ::SEC3-GFP-HIS5*	This study
CGY012	RJD863 *sec3Δ::SEC3-GFP-HIS5 mid2Δ::KAN*^{R}	This study
CGY013	RJD863 *sec3Δ::SEC3-GFP-HIS5 wsc1Δ::LEU2*^{Kl}	This study
CGY015	RJD863 *sec3Δ::SEC3-GFP-HIS5 spa2Δ::LEU2*^{Kl}	This study
CGY016	RJD863 *sec3Δ::SEC3-GFP-HIS5 spa2Δ::LEU2*^{Kl} *mid2Δ::KAN*^{R}	This study
CGY017	RJD863 *sec3Δ::SEC3-GFP-HIS5 spa2Δ::LEU2*^{Kl} *wsc1Δ::URA3*^{Kl}	This study

Table B:

Parameter	Description	Value
P	Turgor pressure of budding yeast	0.6 ± 0.2 MPa [1]
ρ_w	Density of 1,3-β glucans in cell wall	–
μ_0	Apical viscosity of cell wall	–
m_w	Mass of 1,3-β glucan monomer	–
ρ_0	Density of Fks1/2 enzymes in vesicle	–
k_p	Extrusion rate of 1,3-β glucan monomers	–
λ_X	Exocytosis length-scale	$0.6 \pm 0.1\mu$m [2]
		$0.45 \pm 0.10\mu$m [3]
λ_D	Endocytosis length-scale	$1.05 \pm 0.18\mu$m [3]
k_X^0	Apical rate of exocytosis	$0.045s^{-1}$ [4]
k_D^0	Apical rate of endocytosis	$0.02 \pm 0.02s^{-1}$ [3]
		$0.027s^{-1}$ [4]
k_{off}	Inactivation rate of Fks1/2	–

References

[1] Schaber J, Angel Adrover M, Eriksson E, Pelet S, Petelenz-Kurdziel E, Klein D et al. Biophysical properties of *Saccharomyces cerevisiae* and their relationship with HOG pathway activation. Eur Biophys J. 2010; 39(11):1547–1556.

[2] This manuscript

[3] Jose M, Tollis S, Nair D, Sibarita J, McCusker D. Robust polarity establishment occurs via an endocytosis-based cortical corralling mechanism. The Journal of Cell Biology. 2013; 200(4): 407–418.

[4] Carrillo L, Cucu B, Bandmann V, Homann U, Hertel B, Hillmer S, et al. High-Resolution Membrane Capacitance Measurements for Studying Endocytosis and Exocytosis in Yeast. Traffic. 2015; 16(7): 760–772.
